# Deep Learning Model for Grading Metastatic Epidural Spinal Cord Compression on Staging CT

**DOI:** 10.3390/cancers14133219

**Published:** 2022-06-30

**Authors:** James Thomas Patrick Decourcy Hallinan, Lei Zhu, Wenqiao Zhang, Tricia Kuah, Desmond Shi Wei Lim, Xi Zhen Low, Amanda J. L. Cheng, Sterling Ellis Eide, Han Yang Ong, Faimee Erwan Muhamat Nor, Ahmed Mohamed Alsooreti, Mona I. AlMuhaish, Kuan Yuen Yeong, Ee Chin Teo, Nesaretnam Barr Kumarakulasinghe, Qai Ven Yap, Yiong Huak Chan, Shuxun Lin, Jiong Hao Tan, Naresh Kumar, Balamurugan A. Vellayappan, Beng Chin Ooi, Swee Tian Quek, Andrew Makmur

**Affiliations:** 1 Department of Diagnostic Imaging, National University Hospital, 5 Lower Kent Ridge Road, Singapore 119074, Singapore; kuahtricia93@gmail.com (T.K.); desmond_lim@nuhs.edu.sg (D.S.W.L.); xi_zhen_low@nuhs.edu.sg (X.Z.L.); amanda_joanne_cheng@nuhs.edu.sg (A.J.L.C.); sterling_ellis_eide@nuhs.edu.sg (S.E.E.); han_yang_ong@nuhs.edu.sg (H.Y.O.); faimee.erwan@gmail.com (F.E.M.N.); ahmed.alsoorti@gmail.com (A.M.A.); mialmuhaish@gmail.com (M.I.A.); ee_chin_teo@nuhs.edu.sg (E.C.T.); swee_tian_quek@nuhs.edu.sg (S.T.Q.); andrew_makmur@nuhs.edu.sg (A.M.); 2 Department of Diagnostic Radiology, Yong Loo Lin School of Medicine, National University of Singapore, 10 Medical Drive, Singapore 117597, Singapore; 3 Integrative Sciences and Engineering Programme, NUS Graduate School, National University of Singapore, 21 Lower Kent Ridge Road, Singapore 119077, Singapore; e0203764@u.nus.edu; 4 Department of Computer Science, School of Computing, National University of Singapore, 13 Computing Drive, Singapore 117417, Singapore; wenqiao@nus.edu.sg (W.Z.); ooibc@comp.nus.edu.sg (B.C.O.); 5 Department of Diagnostic Imaging, Salmaniya Medical Complex, Rd No 2904, Manama 323, Bahrain; 6 Department of Radiology, Imam Abdulrahman Bin Faisal University, PO BOX 1982, Dammam 31441, Saudi Arabia; 7 Department of Radiology, Ng Teng Fong General Hospital, 1 Jurong East Street 21, Singapore 609606, Singapore; kuan_yuen_yeong@nuhs.edu.sg; 8 National University Cancer Institute, NUH Medical Centre (NUHMC), Levels 8–10, 5 Lower Kent Ridge Road, Singapore 119074, Singapore; kumarakulasinghe_nesaretnam@nuhs.edu.sg; 9 Biostatistics Unit, Yong Loo Lin School of Medicine, National University of Singapore, 10 Medical Drive, Singapore 117597, Singapore; qaiven@nus.edu.sg (Q.V.Y.); medcyh@nus.edu.sg (Y.H.C.); 10 Division of Spine Surgery, Department of Orthopaedic Surgery, Ng Teng Fong General Hospital, 1 Jurong East Street 21, Singapore 609606, Singapore; shuxun_lin@nuhs.edu.sg; 11 University Spine Centre, Department of Orthopaedic Surgery, National University Health System, 1E, Lower Kent Ridge Road, Singapore 119228, Singapore; jonathan_jh_tan@nuhs.edu.sg (J.H.T.); dosksn@nus.edu.sg (N.K.); 12 Department of Radiation Oncology, National University Cancer Institute Singapore, National University Hospital, Singapore 119074, Singapore; bala_vellayappan@nuhs.edu.sg

**Keywords:** deep learning model, metastatic spinal cord compression, metastatic epidural spinal cord compression, CT, MRI, Bilsky classification, spinal metastases classification, spinal metastatic disease, epidural spinal cord compression

## Abstract

**Simple Summary:**

Metastatic epidural spinal cord compression (MESCC) is a disastrous complication of advanced malignancy, and early diagnosis is important to prevent irreversible neurological injury. MRI is the mainstay of diagnosis for MESCC, but it is expensive, and routine screening of asymptomatic patients is not feasible. Staging CT studies are performed routinely as part of the cancer diagnosis and represent an opportunity for earlier diagnosis and treatment planning. In this study, we trained deep learning models for automatic MESCC classification on staging CT studies using spine MRI and manual radiologist labels as the reference standard. On a test set, the DL models showed almost-perfect interobserver agreement for the classification of CT spine images into normal, low, and high-grade MESCC, with kappas ranging from 0.873–0.911 (*p* < 0.001). The DL models (lowest κ = 0.873, 95% CI 0.858–0.887) also showed superior interobserver agreement compared to two radiologists, including a specialist (κ = 0.820, 95% CI 0.803–0.837) and general radiologist (κ = 0.726, 95% CI 0.706–0.747), both *p* < 0.001.

**Abstract:**

Background: Metastatic epidural spinal cord compression (MESCC) is a disastrous complication of advanced malignancy. Deep learning (DL) models for automatic MESCC classification on staging CT were developed to aid earlier diagnosis. Methods: This retrospective study included 444 CT staging studies from 185 patients with suspected MESCC who underwent MRI spine studies within 60 days of the CT studies. The DL model training/validation dataset consisted of 316/358 (88%) and the test set of 42/358 (12%) CT studies. Training/validation and test datasets were labeled in consensus by two subspecialized radiologists (6 and 11-years-experience) using the MRI studies as the reference standard. Test sets were labeled by the developed DL models and four radiologists (2–7 years of experience) for comparison. Results: DL models showed almost-perfect interobserver agreement for classification of CT spine images into normal, low, and high-grade MESCC, with kappas ranging from 0.873–0.911 (*p* < 0.001). The DL models (lowest κ = 0.873, 95% CI 0.858–0.887) also showed superior interobserver agreement compared to two of the four radiologists for three-class classification, including a specialist (κ = 0.820, 95% CI 0.803–0.837) and general radiologist (κ = 0.726, 95% CI 0.706–0.747), both *p* < 0.001. Conclusion: DL models for the MESCC classification on a CT showed comparable to superior interobserver agreement to radiologists and could be used to aid earlier diagnosis.

## 1. Introduction

The spinal column is the most common site for metastases, and spinal metastases affect up to 40% of cancer patients [1]. Spinal metastases can lead to pain and potentially devastating complications, including metastatic epidural spinal cord compression (MESCC), which can lead to paralysis and bowel and bladder dysfunction. Permanent neurological dysfunction in MESCC can be prevented if the condition is detected early and confirmed on imaging, allowing appropriate treatment to be initiated. Unfortunately, if the presentation of MESCC is delayed and the patient is non-ambulatory at presentation, it is unlikely that they will regain the ability to walk [2,3].

To aid in the earlier diagnosis of MESCC, cancer clinicians, patients, and their families need to be aware of the early signs and symptoms of MESCC. Symptoms preceding neurological dysfunction in patients with MESCC include pain (most common and can involve a change in the characteristics of existing pain), altered sensations/paresthesias (e.g., numbness and tingling), and reduced ambulation. However, these symptoms can be subtle and non-specific, especially in those with opioid analgesics, and can overlap with existing pain from spine degeneration. In addition, patients may be asymptomatic in the early stages of MESCC, which in combination with the non-specific symptoms, means that a definitive diagnosis of MESCC with imaging can be delayed [4].

Prior studies and the UK NICE guidelines on metastatic cord compression in adults (2008) advise that an MRI is the initial imaging of choice for the accurate diagnosis of MESCC [5,6]. An MRI provides a detailed assessment of both osseous and soft tissue metastases along the spinal canal and can assess the degree of MESCC and the status of the spinal cord [7]. MESCC is typically classified using the Spine Oncology Study Group (Bilsky) grading scale [8]. The grading scale is important for treatment planning and consists of a six-point classification, which can be subdivided into two key groups. Low-grade disease (0, 1a, and 1b grades) can be targeted for initial radiotherapy (e.g., external beam radiotherapy or stereotactic radiosurgery) and high-grade disease (1c, 2, and 3 grades) with contact or compression of the spinal cord can be considered for initial surgical decompression and subsequent radiotherapy [9].

If an MRI is contraindicated, then a CT myelography, a more invasive procedure, can be considered for the diagnosis of MESCC. Conventional contrast-enhanced CT is another potential option for MESCC diagnosis. It is performed at frequent intervals in cancer patients to stage the overall cancer burden and assess response to treatment [10]. Staging CT studies present an opportunity for earlier diagnosis of MESCC in asymptomatic patients or those with unclear symptomatology or pre-existing back pain. If there is suspicion of MESCC on the staging CT, then a detailed clinical assessment can be undertaken, and a definitive MRI performed. Currently, small studies have shown the utility of a staging CT versus an MRI for the diagnosis of MESCC. Crocker et al. (2011) looked at 41 patients with suspected MESCC and reported a sensitivity of 89% and a specificity of 92% for the detection of MESCC when compared to a subsequent MRI [11].

A deep learning (DL) tool for automatic detection of MESCC on a CT could alert the reporting radiologist and clinicians, allowing for an expedited MRI for the confirmation and the treatment planning of MESCC. This could provide earlier treatment to prevent permanent neurological dysfunction and reduce the requirement for health resources. Prior deep learning in CT and MRI of the spine have shown great promise, especially for the detection of lumbar disc herniation [12] and lumbar spinal stenosis [13,14]. Deep learning for the detection and classification of spinal metastases on CT and MRI is in an early phase and mainly focuses on the detection [15], bone segmentation and metastatic burden [16], or the prediction of a metastasis versus a primary tumor type [17]. To our knowledge, no deep learning tool has been trained to assess MESCC using a staging CT.

The main aims of this study are the following:

1. Develop a deep learning model for automatic detection of MESCC on a staging CT. To our knowledge, this has not been done previously and could expedite earlier diagnosis of MESCC and identify suitable patients for initial radiotherapy versus surgical decompression.
2. Model training and testing will be done using reference standard MESCC gradings on staging CT studies provided by experienced radiologists using axial T2-weighted MRI scans (the current gold standard for MESCC evaluation) performed within two months of the CT.
3. Once the deep learning model is trained, the clinical performance of the model will be compared with that of both subspecialized radiologists with experience in reporting advanced spine imaging and general radiologists on a test set.

## 2. Materials and Methods

The study was conducted in accordance with the Declaration of Helsinki and approved by the Institutional Review Board of the National Healthcare Group (NHG), Singapore. A waiver of consent was granted as this was a retrospective study, and minimal risk was involved.

### 2.1. Dataset Preparation

Retrospective extraction and anonymization of the MRI spines and corresponding staging CT studies from patients with spinal metastatic disease and suspicion of thoracic MESCC were performed over a twelve-year period from September 2007 to September 2019 at the National University Hospital, Singapore. Adult patients (≥ 18 years of age) were included, with imaging studies selected across different MRI and CT scanners (Philips, GE, and Siemens). Corresponding MRI and staging CT studies with a time gap of up to two months (sixty days) were included. MRI and CT studies with spinal instrumentation, poor image quality (e.g., motion artifacts), and non-thoracic spine regions were excluded. CT studies with contrast (portal-venous phase) were included, with the exclusion of non-contrast studies. Supplementary Tables S1 and S2 provide details on the CT and MRI scanner types and parameters, respectively.

The dataset from the National University Hospital, Singapore, was split randomly into 88% for the training/validation set and 12% for the test set. This percentage split is acceptable for deep learning datasets [18].

### 2.2. Dataset Labelling

Imaging data for training were manually reviewed and labeled by two experienced radiologists; a musculoskeletal radiologist (JTPDH; 11-years-experience) and a neuroradiologist (AM; 6-years-experience). Each specialist labeled at least 150 staging CT studies independently. Using a freely available annotation tool (LabelImg—https://github.com/tzutalin/labelImg, accessed on 9 May 2022), bounding boxes were manually deployed at the region of interest (ROI) around the thoracic spinal canal (C7-T1 to the termination of the spinal cord at T12-L3). The cervical region was not evaluated as CT neck studies are not a routine component of our institutional staging CT protocol. Labels were placed on individual axial contrast-enhanced CT images using the corresponding axial T2-weighted MRI images as the reference standard. The axial CT images were provided in three standard Hounsfield unit (HU) window widths (W) and levels (L) for accurate visual assessment; Abdominal soft tissue window (W:400, L:50), spine soft tissue window (W:200, L:100), and bone windows (W:1500, L:300).

**Figure 1:**
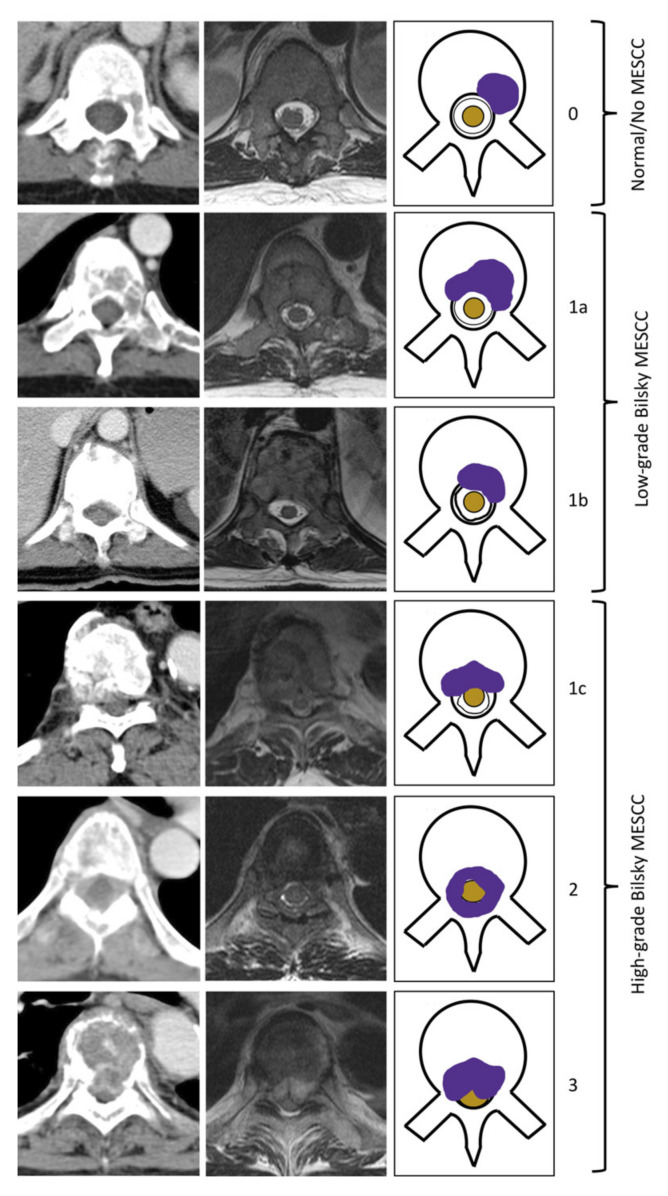
Bilsky grading of metastatic epidural spinal cord compression (MESCC) on MRI and corresponding staging CT of the thoracic spine. Axial contrast-enhanced staging CT studies (portal-venous phase) and corresponding axial T2-weighted (repetition time in msec/echo time in msec, 5300/100) MRI were used. Deep learning model training was performed by radiologists using bounding boxes to highlight the region of interest at each axial CT image. Grade 0/normal: No epidural disease, Grade 1a: Epidural disease with no thecal sac indentation, Grade 1b: Epidural disease with thecal sac indentation, Grade 1c: Epidural disease touching the cord with no displacement, Grade 2: Spinal cord compression with some cerebrospinal fluid (CSF) visible, Grade 3: Spinal cord compression with no CSF visible at the site of compression.

Using bounding boxes, the labeling radiologists graded the MESCC using the Bilsky classification [8]. The Bilsky classification has six categories with low-grade disease (0, 1a, and 1b) potentially treatable with first-line radiotherapy and high-grade disease (1c, 2, and 3) more likely to be treated surgically. A chart of the different Bilsky grades was provided to all labelers (Figure 1). Osteoarthritic findings along the vertebra and disk spaces (e.g., disk herniations, osteophytes, and ligamentum flavum ossification) leading to spinal canal narrowing were labeled and excluded from the analysis [19,20].

The CT test set was labeled using the visual scale and corresponding MRI studies by the specialized radiologist (JTPDH) with 11-years-experience and provided the reference standard. The reference radiologist did not have access to the outputs of the DL model. The DL models were compared against human readers on an internal test set for interobserver variability. The internal test set was labeled independently by subspecialist musculoskeletal radiologists (SEE; 7-years-experience, and FEM; 5-years-experience) and two body radiologists who primarily report staging CT studies (HYO; 2 years, AJLC; 5-years-experience). Prior to labeling, all readers were provided with a visual grading scale and reviewed five practice cases showing a range of low and high-grade Bilsky gradings on corresponding axial MRI and CT images. For the formal assessment, all readers were blinded to the reference standard, DL model gradings, and corresponding MRI studies.

### 2.3. Deep Learning Model Development

**Figure 2:**
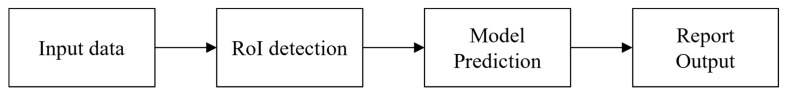
Machine learning pipeline development for both model training and model deployment in a clinical setting. ROI = Region of Interest.

A deep learning pipeline with the sequential region of interest (ROI) detection and classification was developed for automated CT Bilsky analysis. Figure 2 shows our proposed pipeline, which consists of two major steps. First, we employed the Faster R-CNN [21] with the Resnet50 [22] backbone network and fine-tuned this detector on the CT scans to detect the ROIs. Second, multimodal learning models were trained for the ROI classification with the available three different windows, namely the abdominal, bone, and spine windows. In our setting, we argue that each different window can be regarded as one different modality with exclusive characteristics. Therefore, multimodal learning methods were developed to leverage the complementary information from different windows for more accurate analysis [23].

In detail, we developed two multimodal learning methods, called separated window learning (SWL) and combined window learning (CWL). For SWL, we trained one convolutional prototypical network [24] with Resnet50 [25] as its backbone network architecture for each window. Then, we utilized either an average fusion mechanism or a max fusion mechanism at the prediction level to aggregate the complementary information from different windows for the final prediction. In average fusion, we calculate the final prediction probability for each grading as the average of prediction probabilities from the three networks trained in each window. In max fusion, the model predicts the CT Bilsky grading with maximum prediction probability among all the three networks.

**Figure 3:**
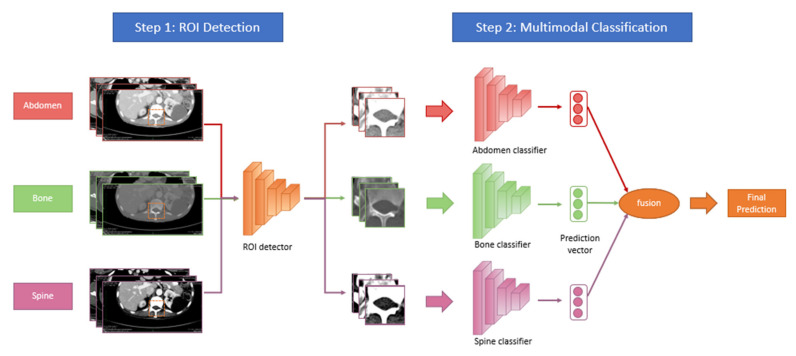
Overview of the developed deep learning pipeline. The developed deep learning pipeline takes input images with information from three different windows. For the first step, a region of interest (ROI) detector is applied to all images of the three windows. In the second step, a window-specific classifier is applied to calculate the prediction probability for each windowed image. We have developed both a separated window learning (SWL) method and a combined window learning (CWL) method. In the separated window learning (SWL) method, three separate networks are trained for the information from three different windows. In the combined window learning (CWL) method, a single network with window-specific batch normalization layers is trained on all windowed images to reduce the memory size. Multimodal fusion techniques, specifically average fusion and max fusion, are applied at the prediction probability vectors to aggregate all the complementary information from images of different windows for the final prediction.

While the SWL method shows the potential of the multimodal learning paradigm, we also found that the separated window training suffers from an undesirable space usage issue due to the large model size. Each window in SWL requires a separated network, and the three different windows result in the tripling of the model size. When there is a memory limit in the final deployment environment (e.g., mobile phone), a smaller model size would always be desirable. To alleviate this issue, we developed combined window learning (CWL). In this method, we shared the weights of the networks trained in different windows apart from the batch normalization layers [26], which are used to mitigate the discrepancy for input CT scans from different windows. Such a design leads to a single network with model-specific batch normalization layers, which keeps the model size almost constant as the batch normalization layers occupy a negligible amount of memory. Finally, we applied either average fusion or max fusion to aggregate all the information from three different windows for the final prediction (Figure 3). The hyper-parameters of our framework are optimized with a hold-out validation set, which is a standard operation in the machine learning community [27].

Finally, one additional benefit of our developed multimodal learning methods is their robustness against missing windowing information. For inputs with incomplete windowing information, our methods can simply aggregate all the available windowing information for the final prediction. All of our models were developed using the Apache SINGA [28] platform and MLCask [29], which is a highly efficient pipeline management system for data analytics, facilitating the management of several versions of the developed algorithms. The code of the deep learning model (SpineAI@NUHS-NUS) is available at https://github.com/NUHS-NUS-SpineAI/SpineAI-Bilsky-Grading-CT, accessed on 9 May 2022.

### 2.4. Statistical Analysis

All statistical analyses were carried out using version 16 (StataCorp LLC, College Station, TX, USA), with significance placed at a 2-sided *p* < 0.05. Assuming that kappa of approximately 0.9 is to be obtained, a minimum of 138 studies (staging CTs) were needed to provide a 95% confidence interval width of 0.1. Over the 12-year study period, a more than sufficient sample of 185 subjects with 444 CT studies was obtained. Descriptive statistics for categorical variables were presented as n (%) and the mean ± standard deviation (range) for continuous variables. Inter-observer agreement for trichotomous (normal, low, and high-grade) and dichotomous (normal/low versus high-grade, and normal versus low/high-grade) Bilsky gradings were assessed with Gwet’s kappa to take into account the high proportion of normal gradings. Sensitivity, specificity, and AUCs were also presented for the two groups of dichotomous Bilsky gradings only.

Levels of agreement were defined for Gwet’s kappa as follows: <0 = poor, 0–0.2 = slight, 0.21–0.4 = fair, 0.41–0.6 = moderate, 0.61–0.8 = substantial, and 0.81–1 = almost-perfect agreement. Further, 95% confidence intervals (CIs) were calculated.

## 3. Results

### 3.1. Patient Characteristics in Datasets

Data collection over the 12-year study period identified 185 patients with 444 CT studies and corresponding MRI spines. Of the 444 CT studies, 86 were excluded as they were either non-contrast (no intravenous contrast administered in 28/86, 32.6%), covered only the cervical or lumbosacral region (49/86, 57.0%), instrumentation was present (4/86, 4.7%), had an interval greater than 60 days between the CT and the MRI (3/86, 3.5%), or were of poor image quality (2/86, 2.3%). Overall, 358 CT staging studies from 185 patients were available for analysis.

Overall, the mean age of the 185 patients was 60 ± 12(SD) (range:18–93 years). The patients were more frequently male (96/185 patients, 51.9%), with breast and lung malignancies the most frequent cancer subtypes (86/185 patients, 46.5%). The majority of thoracic MESCC was located at the thoracolumbar border between T11-L3 (75/185 patients, 40.5%). The patient demographics, types of cancer, and distribution of MESCC along the thoracic spine are shown in Table 1.

**Figure 4:**
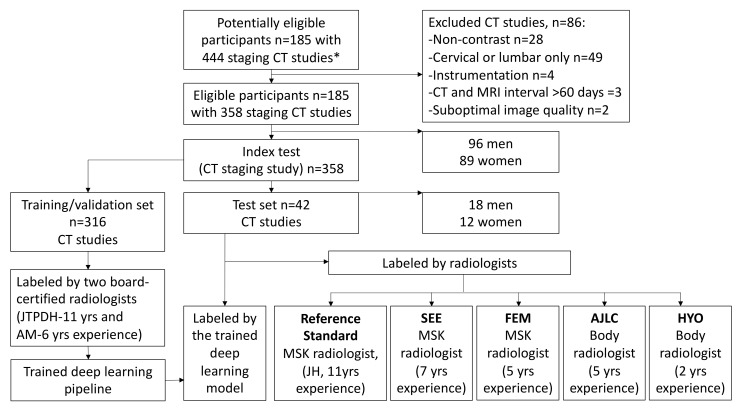
Flow chart of the overall study design and deep learning model development. The model performance was compared with a specialist radiologist (reference standard) and four radiologists. * All CT studies had a corresponding MRI of the thoracic region available. ROI = Region of interest. MSK = musculoskeletal radiologist (specialised in reading spine studies).

The internal imaging data comprising 358 CT staging studies were randomly split into 316 (88%) CTs for training/validation and 42 (12%) CTs for testing, respectively. Figure 4 provides a flow chart of the overall study design.

### 3.2. Reference Standard

The axial images and the CT Bilsky gradings for the internal training/validation and test datasets are shown in Table 2. For the internal training/validation set, there was a predominance of normal or low-grade Bilsky gradings (12071/13400 ROIs, 90.1%), with high-grade Bilsky grading (1c, 2, and 3) accounting for 1329/13400 ROIs (9.9%). In the testing data, high-grade Bilsky grading (1c/2/3) comprised 203/2735 ROIs (7.4%) with a predominance of either normal or low-grade disease (2532/2735 ROIs, 92.6%) at the remaining sites.

### 3.3. Trichotomous Bilsky Classification

On the test set, all versions of the DL model showed almost perfect agreement (kappa range 0.873–0.911, all *p* < 0.001) for trichotomous Bilsky classification (normal, low, and high-grade) (Table 3). The CWL average fusion model showed the highest kappa of 0.911 (95% CI 0.899–0.923), which was superior to the lowest kappa for the SWL spine window (κ = 0.873, 95% CI 0.858–0.887), *p* < 0.001. There was no significant difference in interobserver agreement between the average fusion models using the CWL or SWL methods with kappas of 0.911 (95% CI 0.899–0.923) and 0.904 (95% CI 0.892–0.917), respectively, *p* = 0.484. In comparison to the readers, all versions of the DL model, including the SWL spine window model (κ = 0.873, 95% CI 0.858–0.887) showed superior interobserver agreement compared to the specialist FEM (κ = 0.820, 95% CI 0.803–0.837) and general radiologist HYO (κ = 0.726, 95% CI 0.706–0.747), both *p* < 0.001. There was no significant difference in interobserver agreement between the best performing model (CWL average fusion, κ = 0.911, 95% CI 0.899–0.923) and the best performing readers including AJLC (κ = 0.907, 95% CI 0.895–0.919) and SEE (κ = 0.907, 95% CI 0.895–0.919), *p* = 0.397 and *p* = 0.451, respectively.

### 3.4. Normal/Low Versus High-Grade Dichotomous Bilsky Classification

All versions of the DL model showed almost perfect agreement for dichotomous normal/low versus high-grade MESCC (Table 3), with kappas ranging from 0.938 (95% CI 0.928–0.949) for the SWL abdomen window to 0.972 (95% CI 0.965–0.979) for the CWL spine window, with a significant difference between the two models, *p* < 0.001. There was no significant difference in interobserver agreement between the best performing model (CWL spine window, κ = 0.972, 95% CI 0.965–0.979) and all the readers, including HYO the best performing reader (κ = 0.975, 95%CI 0.968–0.981), *p* = 0.466.

All versions of the DL model showed high AUCs ranging from 0.953 (95% CI 0.934–0.971) for the SWL spine window to 0.971 (95% CI 0.961–0.981) for the SWL max fusion model, with no significant difference between the models (*p*= 0.746) (Table 4). The best performing model (SWL max fusion model, AUC = 0.971, 95% CI 0.961–0.981) had superior performance compared to all readers including FEM and HYO with the best human performance (AUC = 0.891, 95% CI 0.863–0.918 and AUC = 0.891, 95% CI 0.863–0.919, respectively), both *p* < 0.001. Sensitivities for all model versions (range: 92.6–98.0%) were superior to all the readers (range: 66.5–80.8%), with the lowest model sensitivity (92.6% for SWL spine window, 95% CI 88.1–95.8%) significantly greater than the highest reader sensitivity for FEM (80.8%, 95% CI 74.7–86.0%), *p* < 0.001. The DL models and readers all showed high specificities ranging from 94.8% (95% CI 93.8–95.6%) for the SWL abdomen window to 99.8% for SEE (95% CI 99.5–99.9%).

### 3.5. Normal Versus Low/High-Grade Dichotomous Bilsky Classification

All versions of the DL model showed almost perfect agreement for dichotomous normal versus low/high-grade MESCC (detection of any grade of disease), with kappas ranging from 0.889 (95% CI 0.874–0.905) for the SWL spine window to 0.929 (95% CI 0.917–0.941) for both the CWL abdomen window and average fusion methods, with a significant difference between SWL spine window and both the CWL abdomen window and average fusion methods (both *p* < 0.001) (Table 3). The best performing models (CWL abdomen window and average fusion methods, κ = 0.929 (95% CI 0.917–0.941) showed superior interobserver agreement compared to the specialist FEM (κ = 0.816, 95% CI 0.796–0.836) and general radiologist HYO (κ = 0.683, 95% CI 0.656–0.710), both *p* < 0.001. There was no significant difference between the best performing models (CWL abdomen window and average fusion methods, κ = 0.929 (95% CI 0.917–0.941) compared to the best performing specialist reader SEE (κ = 0.928, 95% CI 0.916–0.940), *p*= 0.636).

All versions of the DL model showed high AUCs ranging from 0.899 (95% CI 0.881–0.918) for the CWL abdomen window to 0.924 (95% CI 0.910–0.938) for the SWL Spine-window with no significant differences (*p* = 0.451) (Table 5). All SWL models showed superior AUC compared to all readers, including FEM with the highest AUC of 0.866 (95% CI 0.848–0.885), all *p* < 0.001. Sensitivities for all model versions (range: 83.0–92.7%) were superior to two of the readers AJLC (58.5%, 95% CI 53.6–63.3%), and SEE (68.4%, 95% CI 63.7–72.9%), all *p* < 0.001. The highest DL model sensitivity of 92.7% (SWL spine window, 95% CI 89.7–95.0%) showed no evidence of a difference to HYO, with the highest reader sensitivity of 89.3% (95% CI 85.9–92.1%), *p* = 0.094. All versions of the DL model showed high specificities ranging from 92.2 to 96.8%. All model versions showed higher specificities compared to two of the human readers, with the lowest SWL spine window specificity of 92.2% (95% CI 91.0–93.2%) greater compared to FEM (87.6%, 95% CI 86.2–88.9%) and HYO (78%, 95% CI 76.3–79.7%), both *p* < 0.001.

## 4. Discussion

MESCC is a potentially devastating complication of advanced cancer, and early diagnosis is important to prevent irreversible neurological injury. An MRI is the mainstay of diagnosis for MESCC, but it is expensive and routine screening of asymptomatic patients is not feasible. Staging CT studies are performed routinely as part of the cancer diagnosis and treatment follow-up. Over 62 million CT studies are performed annually in the USA, and a large proportion are staging CT studies [30]. Detection of MESCC on staging CT represents an opportunity for earlier diagnosis, initiation of less invasive treatment (e.g., radiotherapy and systemic therapy), and prevention of permanent neurological dysfunction. In this study, we trained several DL models for automatic MESCC Bilsky grading on staging CT studies using corresponding MRI spine images and manual radiologist labels. On a test dataset, the DL models showed almost-perfect interobserver agreement for trichotomous Bilsky classification (normal, low, and high-grade), with kappas ranging from 0.873–0.911 (*p* < 0.001). All versions of the DL model (lowest κ = 0.873, 95% CI 0.858–0.887) showed superior interobserver agreement compared to the specialist FEM (κ = 0.820, 95% CI 0.803–0.837) and general radiologist HYO (κ = 0.726, 95% CI 0.706–0.747), both *p* < 0.001.

The DL models also showed almost perfect interobserver agreement for both dichotomous groupings, with kappas of 0.938–0.972 (*p* < 0.001) for high-grade Bilsky MESCC classification and kappas of 0.889–0.929 (*p* < 0.001) for any grade of MESCC. For distinction of any grade of MESCC, the best performing DL model (κ = 0.929, 95% CI 0.917–0.941) showed superior interobserver agreement compared to the specialist FEM (κ = 0.816, 95% CI 0.796–0.836) and general radiologist HYO (κ = 0.683, 95% CI 0.656–0.710), both *p* < 0.001. The DL models showed superior sensitivities (range = 92.6–98.0%) to all readers for the classification of high-grade Bilsky disease, which is important to ensure that this time-critical disease is detected early and appropriate treatment is planned. For detection of any grade of MESCC, the DL models showed high sensitivities (range = 83.0–92.7%) and specificities (range = 92.2–96.8%).

When comparing the CWL and SWL methods, there was no clear improvement in the interobserver agreement or AUCs with the more memory-space-intensive SWL method. Fusion (Max or average) for both the CWL and SWL methods showed comparable or marginal increased interobserver agreement and AUCs compared to the single window models. However, multimodal fusion methods are still preferred as they are robust when faced with missing windowing information. They can simply aggregate all the available windowing information for the final prediction.

Deep learning is being used to aid the imaging diagnosis of many different conditions, including liver segmentation [31] and vertebral segmentation for evaluation of the spine [32]. Other deep learning applications in spinal conditions include degenerative lumbar spinal stenosis on an MRI [13], automated segmentation of the spinal cord for radiotherapy planning [33], and prediction of treatment outcomes and complications in spinal oncology [34]. Deep learning for automated detection of spinal canal compromise and spinal cord compression has been investigated on MRI. Merali et al. (2021) trained a deep learning algorithm to detect compression of the spinal cord on MRI of the cervical spine due to spondylosis [35]. Overall, 201 surgical patients were assessed, and their deep learning model had 88% sensitivity, 89% specificity, and an AUC of 0.94. Most recently, Hallinan et al. (2022) developed a deep learning model for the prediction of low and high-grade Bilsky classification on MRI of the thoracic spine. On internal and external testing, the DL model showed high agreement (κ = 0.92–0.94, *p* < 0.001) for two-grade Bilsky classification, which was similar to specialist labelers (κ = 0.95–0.98, all *p* < 0.001), including a spine surgeon, a radiation oncologist, and a radiologist [36].

The developed CT Bilsky DL model could improve the care of patients with complications arising from spinal metastases. Firstly, CT studies with high-grade MESCC could be triaged for expedited radiologist review and definitive MRI, which is important for accurate surgical and/or radiotherapy planning. This would also provide a window of opportunity to institute treatment before neurological deficits set in, especially in patients with unclear symptoms, e.g., back pain masked by analgesia. In addition, CT studies with no or low-grade Bilsky disease could be managed without requiring an MRI, which could save on healthcare costs. It should be emphasized that the CT DL model would only be an adjunct to the clinical radiologist and multidisciplinary team. MESCC management is not only dependent on imaging features and relies on clinical presentation, including mechanical pain and neurological dysfunction.

Our study has several limitations. Firstly, we assessed the Bilsky classification of MESCC on a CT scan using axial portal-venous images. We did not have the original source images, coronal or sagittal reconstructions available for all CT scans over the study period. The use of multiplanar images (especially sagittal images) could further improve the radiologist and DL model performance. Secondly, CT scans with a corresponding MRI within two months were analyzed. This may have allowed for interval progression of the disease in the interim. Thirdly, the CT scans were obtained from a database of patients with known MESCC with a high proportion of positive cases with epidural disease. Testing the model on external datasets will be useful to ensure the model is generalizable. Fourth, we used two-grade Bilsky MESCC classification with Bilsky grade 1c designated as high-grade MESCC. This is contentious, as individuals with Bilsky grade 1c do not typically have neurological dysfunction requiring expedited surgical intervention. Fifth, we did not evaluate the accuracy of the Bilsky MESCC classification along the cervical spine. Future work could assess the utility of the model on the cervical region, but this would involve collecting a CT neck dataset. Finally, labeling the CT images for DL model training was a highly-supervised, labor-intensive process. Subsequently, more comprehensive datasets could use semi-supervised learning, which reduces the data annotation burden by leveraging unlabeled data to boost the DL model performance [37,38,39].

## 5. Conclusions

In conclusion, we developed a deep learning (DL) model for the Bilsky grading of metastatic epidural spinal cord compression on staging CT studies. The deep learning model had a superior interobserver agreement for the detection of trichotomous Bilsky grading (normal, low, and high-grade) compared to general and specialist radiologists. The deep learning model could be used to triage patients for an urgent MRI if there is high-grade disease or reduce the need for an MRI when there is no evidence of high-grade disease on CT. Future work using our CT MESCC deep learning model would involve adding clinical data (e.g., cancer subtype, neurological impairment, and pre-existing medical conditions) to improve the selection of patients for more aggressive therapy, including stereotactic body radiotherapy (SBRT) and surgery [40]. Additional work would also involve the prospective deployment of the deep learning model onto the institutional radiology reporting system. Implementation can be challenging as dedicated IT infrastructure is required. A user-friendly seamless integration into the daily workflow will be essential to ensure radiologists utilize the model without limitations [41].

## Figures and Tables

**Table 1:** Patient demographical data and characteristics for the deep learning model training/validation and test datasets.

Characteristics	Internal Training/Validation Set (*n* = 155)	Internal Test Set (*n* = 30)
Age (years) *	60 ± 12.1 (18–93)	58 ± 11.6 (32–76)
Women	77 (49.7)	12 (40.0)
Men	78 (50.3)	18 (60.0)
Cancer Subtype		
Lung	36 (23.2)	8 (26.7)
Breast	33 (21.3)	9 (30.0)
Colon	15 (9.7)	3 (10.0)
Prostate	13 (8.4)	0 (0)
Renal cell carcinoma	12 (7.7)	2 (6.7)
Multiple Myeloma	10 (6.5)	1 (3.3)
Hepatocellular carcinoma	8 (5.2)	1 (3.3)
Nasopharyngeal carcinoma	6 (3.9)	0 (0)
Others	22 (14.2)	6 (20.0)
No. of staging CT thoracic studies	316/358 (88)	42/358 (12)
MESCC location		
Diffuse thoracic ^#^	49 (15.5)	8 (19.0)
C7-T2	21 (6.6)	2 (4.8)
T3-T10	97 (30.7)	14 (33.3)
T11-L3	128 (40.5)	15 (35.7)
No epidural disease	21 (6.6)	3 (7.1)

Note- MESCC = Malignant epidural spinal cord compression. * The values are mean ± SD (range) for numerical variables and n (%) for categorical variables. # Two or greater sites of thoracic epidural disease.

**Table 2:** Reference standard Bilsky grades for metastatic epidural spinal cord compression.

MESCC Grade on CT	Internal Training/Validation Set	Internal Test Set
Normal/Bilsky 0	10,594 (79.1%)	2323 (84.9%)
Low-grade Bilsky (1a, 1b)	1477 (11.0%)	209 (7.6%)
High-grade Bilsky (1c, 2, 3)	1329 (9.9%)	203 (7.4%)
Totals	13,400	2735

Note- Values are numbers (%). A region of interest (bounding box) for Bilsky grade was drawn at each axial contrast-enhanced CT image using axial T2-weighted MRI as a reference standard. MESCC = Malignant Epidural Spinal Cord Compression.

**Table 3:** Internal test set classifications using trichotomous and dichotomous Bilsky gradings on staging CT.

	Trichotomous Grading		Dichotomous Grading			
	Normal, Low and High		Normal/Low vs. High		Normal vs. Low/High	
Reader	Kappa (95% CI)	*p*-Value	Kappa (95% CI)	*p*-Value	Kappa (95% CI)	*p*-Value
AJLC	0.907 (0.895–0.919)	<0.001	0.960 (0.952–0.968)	<0.001	0.915 (0.903–0.928)	<0.001
SEE	0.907 (0.895–0.919)	<0.001	0.963 (0.956–0.971)	<0.001	0.928 (0.916–0.940)	<0.001
FEM	0.820 (0.803–0.837)	<0.001	0.954 (0.945–0.963)	<0.001	0.816 (0.796–0.836)	<0.001
HYO	0.726 (0.706–0.747)	<0.001	0.975 (0.968–0.981)	<0.001	0.683 (0.656–0.710)	<0.001
Combined method						
Abdomen-window	0.891 (0.878–0.904)	<0.001	0.966 (0.959–0.974)	<0.001	0.929 (0.917–0.941)	<0.001
Bone-window	0.903 (0.891–0.916)	<0.001	0.965 (0.957–0.972)	<0.001	0.901 (0.887–0.915)	<0.001
Spine-window	0.901 (0.888–0.914)	<0.001	0.972 (0.965–0.979)	<0.001	0.927 (0.915–0.939)	<0.001
Max Fusion-1	0.909 (0.896–0.921)	<0.001	0.968 (0.961–0.975)	<0.001	0.919 (0.906–0.932)	<0.001
Average Fusion-1	0.911 (0.899–0.923)	<0.001	0.968 (0.961–0.975)	<0.001	0.929 (0.917–0.941)	<0.001
Separated method						
Abdomen-window	0.885 (0.871–0.899)	<0.001	0.938 (0.928–0.949)	<0.001	0.914 (0.900–0.927)	<0.001
Bone-window	0.897 (0.884–0.910)	<0.001	0.953 (0.944–0.962)	<0.001	0.908 (0.894–0.921)	<0.001
Spine-window	0.873 (0.858–0.887)	<0.001	0.971 (0.964–0.978)	<0.001	0.889 (0.874–0.905)	<0.001
Max Fusion	0.891 (0.878–0.905)	<0.001	0.956 (0.947–0.965)	<0.001	0.915 (0.901–0.928)	<0.001
Average Fusion	0.904 (0.892–0.917)	<0.001	0.962 (0.954–0.970)	<0.001	0.923 (0.910–0.935)	<0.001

**Table 4:** Internal test set sensitivity, specificity, and AUCs for the DL model and radiologists using dichotomous Bilsky grading (normal/low versus high) on CT.

Reader	Sensitivity (95% CI)	Specificity (95% CI)	AUC (95% CI)
AJLC	66.5 (59.6–73.0)	98.9 (98.5–99.3)	0.827 (0.795–0.860)
SEE	59.1 (52.0–65.9)	99.8 (99.5–99.9)	0.794 (0.760–0.828)
FEM	80.8 (74.7–86.0)	97.3 (96.6–97.9)	0.891 (0.863–0.918)
HYO	78.8 (72.5–84.2)	99.3 (98.9–99.6)	0.891 (0.863–0.919)
Combined method			
Abdomen-window	96.6 (93.0–98.6)	97.2 (96.5–97.8)	0.969 (0.956–0.982)
Bone-window	95.6 (91.8–98.0)	97.1 (96.4–97.8)	0.964 (0.949–0.978)
Spine-window	95.1 (91.1–97.6)	97.8 (97.2–98.4)	0.965 (0.949–0.980)
Max Fusion-1	96.6 (93.0–98.6)	97.4 (96.7–97.9)	0.970 (0.957–0.982)
Average Fusion-1	96.6 (93.0–98.6)	97.4 (96.7–97.9)	0.970 (0.957–0.982)
Separated method			
Abdomen-window	96.6 (93.0–98.6)	94.8 (93.8–95.6)	0.957 (0.943–0.970)
Bone-window	97.0 (93.7–98.9)	96.0 (95.1–96.7)	0.965 (0.953–0.978)
Spine-window	92.6 (88.1–95.8)	97.9 (97.3–98.4)	0.953 (0.934–0.971)
Max Fusion-1	98.0 (95.0–99.5)	96.2 (95.3–96.9)	0.971 (0.961–0.981)
Average Fusion-1	95.6 (91.8–98.0)	96.9 (96.1–97.5)	0.962 (0.948–0.977)

**Table 5:** Internal test set sensitivity, specificity, and AUCs for the DL model and radiologists using dichotomous Bilsky gradings (normal versus low/high) on CT.

Reader	Sensitivity (95% CI)	Specificity (95% CI)	AUC (95% CI)
AJLC	58.5 (53.6–63.3)	99.5 (99.2–99.8)	0.790 (0.766–0.814)
SEE	68.4 (63.7–72.9)	99.1 (98.6–99.4)	0.838 (0.815–0.860)
FEM	85.7 (81.9–88.9)	87.6 (86.2–88.9)	0.866 (0.848–0.885)
HYO	89.3 (85.9–92.1)	78.0 (76.3–79.7)	0.837 (0.820–0.854)
Combined method			
Abdomen-window	83.0 (79.0–86.5)	96.8 (96.0–97.5)	0.899 (0.881–0.918)
Bone-window	88.3 (84.7–91.2)	93.8 (92.7–94.7)	0.910 (0.894–0.927)
Spine-window	84.1 (80.2–87.5)	96.5 (95.7–97.2)	0.903 (0.885–0.921)
Max Fusion-1	87.1 (83.5–90.2)	95.3 (94.4–96.1)	0.912 (0.895–0.929)
Average Fusion-1	85.2 (81.4–88.5)	96.4 (95.6–97.1)	0.908 (0.891–0.926)
Separated method			
Abdomen-window	89.1 (85.7–91.9)	94.6 (93.6–95.5)	0.918 (0.903–0.934)
Bone-window	89.0 (85.6–91.9)	94.2 (93.1–95.1)	0.916 (0.900–0.932)
Spine-window	92.7 (89.7–95.0)	92.2 (91.0–93.2)	0.924 (0.910–0.938)
Max Fusion-1	89.6 (86.2–92.3)	94.6 (93.6–95.5)	0.921 (0.905–0.936)
Average Fusion-1	89.3 (85.9–92.1)	95.3 (94.3–96.1)	0.923 (0.907–0.938)

## Data Availability

The deep learning model (SpineAI@NUHS-NUS) code is available at https://github.com/NUHS-NUS-SpineAI/SpineAI-Bilsky-Grading-CT (accessed on 9 May 2022).

## Supplementary Materials

The following supporting information can be downloaded at: https://www.mdpi.com/article/10.3390/cancers14133219/s1, Table S1: CT Platform and parameters; Table S2: MRI Platforms and parameters for axial T2-weighted Imaging (Reference standard).
