# Supplementary material for: Deep Learning Model for Grading Metastatic Epidural Spinal Cord Compression on Staging CT
Source: Cancers (Basel). 2022 Jun 30;14(13):3219. doi: 10.3390/cancers14133219 (PMC9264856; doi:10.3390/cancers14133219)
Supplement: Supplementary file 1 [file cancers-14-03219-s001.zip › cancers-1741751-supplementary.pdf]

cancers

MDPI

Supplementary Materials

Deep Learning Model for Grading Metastatic Epidural Spinal Cord Compression on Staging CT

James Thomas Patrick Decourcy Hallinan [1,2,*,†], Lei Zhu [3,4,†], Wenqiao Zhang [4], Tricia Kuah [1], Desmond Shi Wei Lim [1], Xi Zhen Low [1], Amanda J. L. Cheng [1,2], Sterling Ellis Eide [1,2], Han Yang Ong [1,2], Faimee Erwan Muhamat Nor [1,2], Ahmed Mohamed Alsooreti [1,5], Mona I. AlMuhaish [1,6], Kuan Yuen Yeong [7], Ee Chin Teo [1], Nesaretnam Barr Kumarakulasinghe [8], Qai Ven Yap [9], Yiong Huak Chan [9], Shuxun Lin [10], Jiong Hao Tan [11], Naresh Kumar [11], Balamurugan A. Vellayappan [12], Beng Chin Ooi [4], Swee Tian Quek [1,2] and Andrew Makmur [1,2]

Table S1. CT Platforms and parameters.

Parameter	Siemens 4-Slice	Siemens 64-Slice	Philips 256-Slice	Siemens 384-Slice	Ge 512-Slice
Pitch	1.5	1.2	0.984	0.8	0.531
Slice thickness (mm)	5	5	3	3	3
Collimation (mm)	4 × 1	32 × 0.6	128 × 0.625	192 × 0.6	256 × 0.625
kV	120	120	100	100	100-120
Reference mAs	180	200	250	200	200
Rotation time (s)	0.5	0.5	0.5	0.5	0.5

Note—kV = Kilovoltage, mAs = Milliampere-seconds, GE = General Electric. All five CT scanners were situated at the National University Hospital, Singapore. All studies were performed in the supine position in a craniocaudal direction. Contrast volume for all CT scanners = 70–100 ml depending on patient size, at a rate of 1.2–1.5 mL/s.

Table S2. MRI Platforms and parameters for axial T2-weighted Imaging (Reference standard).

Parameter	GE 1.5-T	GE 1.5-T	Siemens 1.5-T	GE 3.0-T	Siemens 3.0-T
TR (msec)	3500	3500	4000	5300	5300
TE (msec)	80	80	90	100	100
Slice thickness (mm)	5	5	5	5	5
Gap (mm)	6	6	6	6	6
Field of view (mm^2)	200 × 200	200 × 200	160 × 160	200 × 200	160 × 160
Matrix	512 × 512	512 × 512	320 × 320	512 × 512	640 × 640

Note—TR = repetition time, TE = echo time, GE = General Electric. All scanners were situated at the National University Hospital, Singapore. All scans were conducted in the supine position with a torso coil.
